# Supplementary material for: Hybrid Models and Biological Model Reduction with PyDSTool
Source: PLoS Comput Biol. 2012 Aug 9;8(8):e1002628. doi: 10.1371/journal.pcbi.1002628 (PMC3415397; doi:10.1371/journal.pcbi.1002628)
Supplement: Text S4 — Complete source code for the PyDSTool package (version 0.88.120504). Includes API documentation and help files linking to web pages. This file is identical to the current public release on Sourceforge.net. (ZIP) [file pcbi.1002628.s004.zip › PyDSTool/html/PyDSTool.Trajectory'.Trajectory-class.html]

xml version="1.0" encoding="ascii"?


PyDSTool.Trajectory'.Trajectory


| Home | Trees | Indices | Help | PyDSTool |
| --- | --- | --- | --- | --- |

Package PyDSTool ::
Module Trajectory' ::
Class Trajectory

[hide private]

[frames] | no frames]

# Class Trajectory

source code

```
object --+
         |
        Trajectory
```

Known Subclasses:
:   - HybridTrajectory

---

Parameterized and non-parameterized trajectory class. vals must be a
sequence of variable objects.

Non-parameterized objects are created by implicit function-defined
generators, e.g. ImplicitFnGen.


|  |  |
| --- | --- |
| Instance Methods | [hide private] |

|  |  |
| --- | --- |
| \_\_call\_\_(self, t, coords=None, checklevel=None, asGlobalTime=False, asmap=False)  Evaluate a parameterized trajectory at given independent variable value(s), interpreted as local times by default, unless asGlobalTime==True (default is False, unlike with sample method). | source code |

|  |  |
| --- | --- |
| \_\_copy\_\_(self) | source code |

|  |  |
| --- | --- |
| \_\_deepcopy\_\_(self, memo=None, \_nil=`[``]`) | source code |

|  |  |
| --- | --- |
| \_\_del\_\_(self) | source code |

|  |  |
| --- | --- |
| \_\_getstate\_\_(self) | source code |

|  |  |
| --- | --- |
| \_\_init\_\_(self, name, vals, coordnames=None, modelNames=None, timeInterval=None, modelEventStructs=None, eventTimes=None, events=None, FScompatibleNames=None, FScompatibleNamesInv=None, abseps=None, globalt0=0, checklevel=0, norm=2, parameterized=True)  x.\_\_init\_\_(...) initializes x; see x.\_\_class\_\_.\_\_doc\_\_ for signature | source code |

|  |  |
| --- | --- |
| \_\_repr\_\_(self)  str(x) | source code |

|  |  |
| --- | --- |
| \_\_setstate\_\_(self, state) | source code |

|  |  |
| --- | --- |
| \_\_str\_\_(self)  str(x) | source code |

|  |  |
| --- | --- |
| \_createEventTimes(self) | source code |

|  |  |
| --- | --- |
| \_infostr(self, verbose=1) | source code |

|  |  |
| --- | --- |
| delete\_variables(self, coords)  coords is a list of coordinate names to remove | source code |

|  |  |
| --- | --- |
| getEventTimes(self, evnames=None, asGlobalTime=True)  Returns a list of times at which the named events occurred in global time, unless asGlobalTime option set to False (default is True). | source code |

|  |  |
| --- | --- |
| getEvents(self, evnames=None, asGlobalTime=True)  Returns a pointset of all named events occuring in global time, unless asGlobalTime option set to False (default is True). | source code |

|  |  |
| --- | --- |
| info(self, verboselevel=1) | source code |

|  |  |
| --- | --- |
| mapNames(self, themap)  themap is a symbolMapClass mapping object for remapping coordinate names | source code |

|  |  |
| --- | --- |
| sample(self, coords=None, dt=None, tlo=None, thi=None, doEvents=True, precise=False, asGlobalTime=True)  Uniformly sample the named trajectory over range indicated. | source code |

|  |  |
| --- | --- |
| truncate\_to\_idx(self, idx)  Truncate trajectory according to a last coordinate specified by idx argument, provided trajectory is defined by an underlying mesh. | source code |

|  |  |
| --- | --- |
| truncate\_to\_indepvar(self, t)  Truncate trajectory according to an independent variable value given by t argument, provided trajectory is defined as parameterized. | source code |

|  |  |
| --- | --- |
| underlyingMesh(self, coords=None, FScompat=True)  Return a dictionary of the underlying independent variables` meshes, where they exist. | source code |

**Inherited from `object`**:
`__delattr__`,
`__getattribute__`,
`__hash__`,
`__new__`,
`__reduce__`,
`__reduce_ex__`,
`__setattr__`


|  |  |
| --- | --- |
| Properties | [hide private] |

**Inherited from `object`**:
`__class__`


|  |  |
| --- | --- |
| Method Details | [hide private] |

|  |  |
| --- | --- |
| \_\_call\_\_(self, t, coords=None, checklevel=None, asGlobalTime=False, asmap=False)  *(Call operator)* | source code |

Evaluate a parameterized trajectory at given independent variable
value(s), interpreted as local times by default, unless
asGlobalTime==True (default is False, unlike with sample method).

asmap option allows continuous trajectory to be called as a map, with
t = 0 or 1 to return the two endpoints. Result includes actual time
values (in global time if asGlobalTime==True).

|  |  |
| --- | --- |
| \_\_init\_\_(self, name, vals, coordnames=None, modelNames=None, timeInterval=None, modelEventStructs=None, eventTimes=None, events=None, FScompatibleNames=None, FScompatibleNamesInv=None, abseps=None, globalt0=0, checklevel=0, norm=2, parameterized=True)  *(Constructor)* | source code |

x.\_\_init\_\_(...) initializes x; see x.\_\_class\_\_.\_\_doc\_\_ for
signature

Overrides: object.\_\_init\_\_: *(inherited documentation)*

|  |  |
| --- | --- |
| \_\_repr\_\_(self)  *(Representation operator)* | source code |

str(x)

Overrides: object.\_\_repr\_\_: *(inherited documentation)*

|  |  |
| --- | --- |
| \_\_str\_\_(self)  *(Informal representation operator)* | source code |

str(x)

Overrides: object.\_\_str\_\_: *(inherited documentation)*

|  |  |
| --- | --- |
| getEventTimes(self, evnames=None, asGlobalTime=True) | source code |

Returns a list of times at which the named events occurred in global
time, unless asGlobalTime option set to False (default is True). If no
events are named, all are used.

|  |  |
| --- | --- |
| getEvents(self, evnames=None, asGlobalTime=True) | source code |

Returns a pointset of all named events occuring in global time, unless
asGlobalTime option set to False (default is True). If no events are
named, all are used.

|  |  |
| --- | --- |
| sample(self, coords=None, dt=None, tlo=None, thi=None, doEvents=True, precise=False, asGlobalTime=True) | source code |

```
Uniformly sample the named trajectory over range indicated.
Returns a Pointset.

If doEvents=True (default), the event points for the trajectory
  are included in the output, regardless of the dt sampling rate.

The order of variable names given in the 'coords' argument is ignored.

precise=True causes the trajectory position to be evaluated precisely
  at the t values specified, which will invoke slow interpolation
  (requires dt to be set otherwise an exception will be raised).
precise=False (default) causes the nearest underlying mesh positions
  to be used, if available (otherwise the behaviour is the same as
  precise=True provided dt has been set, otherwise an exception will be
  raised).

If dt is not given, the underlying time mesh is used, if available.
```

|  |  |
| --- | --- |
| underlyingMesh(self, coords=None, FScompat=True) | source code |

Return a dictionary of the underlying independent variables` meshes,
where they exist. (Dictionary contains references to the meshes, not
copies of the meshes.) Always returns data in local time frame.

FScompat option selects whether to use FuncSpec compatible naming for
variables (mainly for internal use).

  


| Home | Trees | Indices | Help | PyDSTool |
| --- | --- | --- | --- | --- |

|  |  |
| --- | --- |
| Generated by Epydoc 3.0.1 on Fri May 4 15:24:10 2012 | http://epydoc.sourceforge.net |
